# Analyzing the Clinical Potential of Stromal Vascular Fraction: A Comprehensive Literature Review

**DOI:** 10.3390/medicina60020221

**Published:** 2024-01-27

**Authors:** Evgeniy Nikolaevich Goncharov, Oleg Aleksandrovich Koval, Eremin Ilya Igorevich, Manuel De Jesus Encarnacion Ramirez, Renat Nurmukhametov, Kotenko Konstantin Valentinovich, Nicola Montemurro

**Affiliations:** 1 Petrovsky Russian Scientific Center of Surgery, 121359 Moscow, Russia; 2 Neurological Surgery, Peoples Friendship University of Russia, 103274 Moscow, Russia; 3 Academician B.V. Petrovsky, Department of Restorative Medicine and Biomedical Technologies, 117997 Moscow, Russia; 4 Department of Neurosurgery, Azienda Ospedaliero Universitaria Pisana (AOUP), 56100 Pisa, Italy

**Keywords:** regenerative medicine, stromal vascular fraction, cell mixture, tissue regeneration, graft survival, surgery

## Abstract

*Background:* Regenerative medicine is evolving with discoveries like the stromal vascular fraction (SVF), a diverse cell group from adipose tissue with therapeutic promise. Originating from fat cell metabolism studies in the 1960s, SVF’s versatility was recognized after demonstrating multipotency. Comprising of cells like pericytes, smooth muscle cells, and, notably, adipose-derived stem cells (ADSCs), SVF offers tissue regeneration and repair through the differentiation and secretion of growth factors. Its therapeutic efficacy is due to these cells’ synergistic action, prompting extensive research. *Methods:* This review analyzed the relevant literature on SVF, covering its composition, action mechanisms, clinical applications, and future directions. An extensive literature search from January 2018 to June 2023 was conducted across databases like PubMed, Embase, etc., using specific keywords. *Results:* The systematic literature search yielded a total of 473 articles. Sixteen articles met the inclusion criteria and were included in the review. This rigorous methodology provides a framework for a thorough and systematic analysis of the existing literature on SVF, offering robust insights into the potential of this important cell population in regenerative medicine. *Conclusions:* Our review reveals the potential of SVF, a heterogeneous cell mixture, as a powerful tool in regenerative medicine. SVF has demonstrated therapeutic efficacy and safety across disciplines, improving pain, tissue regeneration, graft survival, and wound healing while exhibiting immunomodulatory and anti-inflammatory properties.

## 1. Introduction

The field of regenerative medicine is perpetually evolving, constantly being shaped by ground-breaking discoveries that promise to revolutionize the way we approach various medical conditions. One of the key players in this landscape is the stromal vascular fraction (SVF), characterized by its diverse cellular composition extracted from the adipose tissue that has demonstrated significant therapeutic potential across multiple medical disciplines [1].

Understanding the journey of SVF in medicine necessitates a glimpse into its historical context. The 1960s marked the advent of the SVF narrative when Rodbell initiated his studies into fat cell metabolism, a pursuit that ultimately led to the identification of an ‘active’ fraction of non-adipocyte cells, a collective later known as SVF [1]. The term, however, came into mainstream usage only after the pivotal work by Zuk et al., which unearthed the multipotency of adipose-derived stromal cells, a vital component of SVF [2].

SVF are a heterogeneous mixture of cells, pericytes, smooth muscle cells, and, most importantly, adipose-derived stem cells (ADSCs) [1]. These cells play a crucial role in tissue regeneration and repair, primarily due to their ability to differentiate into various cell types and release angiogenic and anti-inflammatory factors [3]. Among these, the ADSCs are particularly notable for their multipotency, enabling them to differentiate into various cell types such as adipocytes, osteoblasts, and chondrocytes under appropriate conditions [4]. Moreover, these cells are known for their angiogenic and immunomodulatory capabilities, primarily due to their secretion of growth factors and cytokines [5]. The therapeutic efficacy of SVF can be attributed to these diverse cell types acting in synergy. While the regenerative and reparative capacities can be traced back to the ADSCs, the immune cells within SVF contribute to the immunomodulatory effects, essential for tissue repair and regeneration. Over the following decades, this recognition spiraled into a flurry of research investigating the regenerative potential of SVF, spurred by its accessibility and abundant stem cell content. SVF began garnering attention across diverse disciplines [6].

SVF has been employed in various clinical settings due to its regenerative, immunomodulatory, and anti-inflammatory properties. In the field of plastic and reconstructive surgery, SVF-enriched fat grafting has been shown to improve graft survival and wound healing [7]. In orthopedics, SVF has been used to treat osteoarthritis, with studies reporting improvement in pain scores and joint function [8]. In cardiology, SVF therapy is being explored for myocardial ischemia, with encouraging results in pre-clinical and early-phase clinical trials [9]. These varied applications underscore the versatile nature of SVF and its potential to revolutionize the landscape of regenerative medicine. However, the current understanding of SVF is not without limitations and challenges, necessitating further investigation to unlock its full therapeutic potential [10].

The objective of this comprehensive review is to meticulously scrutinize the breadth and depth of contemporary clinical literature concerning SVF, from its inception to its present-day applications, spotlighting potential future trajectories. By compiling and critically examining a wide array of studies, we endeavor to offer a panoramic view of SVF’s potential, thereby contributing to the foundational knowledge that propels the field of regenerative medicine forward.

## 2. Materials and Methods

In this review, we employed a systematic approach to gather and analyze the relevant literature concerning SVF. The objective was to provide a comprehensive overview of SVF, including its biological composition, mechanisms of action, clinical applications, and potential future directions.

### 2.1. Search Strategy

An extensive literature search was conducted using several databases: PubMed, Embase, Scopus, Web of Science, and the Cochrane Library. The search period was from January 2018 to June 2023 as this period of time represents the most recent five years, ensuring that the data and research findings are current. This is particularly important in fields where advancements happen rapidly, as older studies might become outdated or less relevant. The primary keywords used were ‘stromal vascular fraction’, ‘adipose-derived stromal cells’, ‘adipose tissue’, ‘regenerative medicine’, and ‘clinical applications’. These keywords were used in combination with other terms relevant to the specific sections of this review. For example, for the section on clinical applications, terms like ‘wound healing’, ‘osteoarthritis’, ‘myocardial ischemia’, etc., were used in conjunction with the primary keywords.

### 2.2. Selection Criteria

Inclusion criteria comprised original research articles. The selected studies included prospective and/or retrospective case series, and review articles written in English that explored the composition, mechanism of action, therapeutic applications, and future perspectives of SVF. Studies that were not peer-reviewed, such as preprints, were excluded. Likewise, articles not available in English, letters to the editor, studies that did not provide explicit data on SVF, and duplicate studies were also omitted from this review. For clinical trials, only those that reported clear methodologies, patient outcomes, and statistical analyses were considered. Articles had to be explicitly centered on the stromal vascular fraction (SVF). This ensured that the study provided specific insights into SVF, rather than a peripheral or broad overview of adipose tissue or regenerative medicine. The paper had to showcase a sound research methodology, which is a testament to the credibility, reliability, and replicability of the study’s findings. Those with ambiguous or poorly defined methodologies were not considered appropriate for inclusion. Research studies with a limited sample size, specifically those with fewer than 5 participants, might not provide the robust evidence that this review aims to collate. Non-original research articles, such as commentaries, editorials, and opinion papers were excluded to maintain the integrity and objective of our study.

### 2.3. Data Extraction

For each selected article, data were extracted by two independent reviewers (ENG and NM). The data comprised the year of publication, study type, the number of participants (for clinical trials), main findings, and conclusions and complications. Any discrepancies between the reviewers were resolved through discussion until a consensus was reached.

## 3. Results

The systematic literature search yielded a total of 787 articles. After removing duplicates and screening titles and abstracts, 84 full-text articles were assessed for eligibility. Of these, 16 articles met the inclusion criteria and were included in the review (Table 1) [11,12,13,14,15,16,17,18,19,20,21,22,23,24,25,26]. The selected studies included prospective and/or retrospective case series, randomized controlled clinical trials, and reviews. This rigorous methodology provides a framework for a thorough and systematic analysis of the existing literature on SVF, offering robust insights into the potential of this important cell population in regenerative medicine.

## 4. Discussion

**Figure 1:**
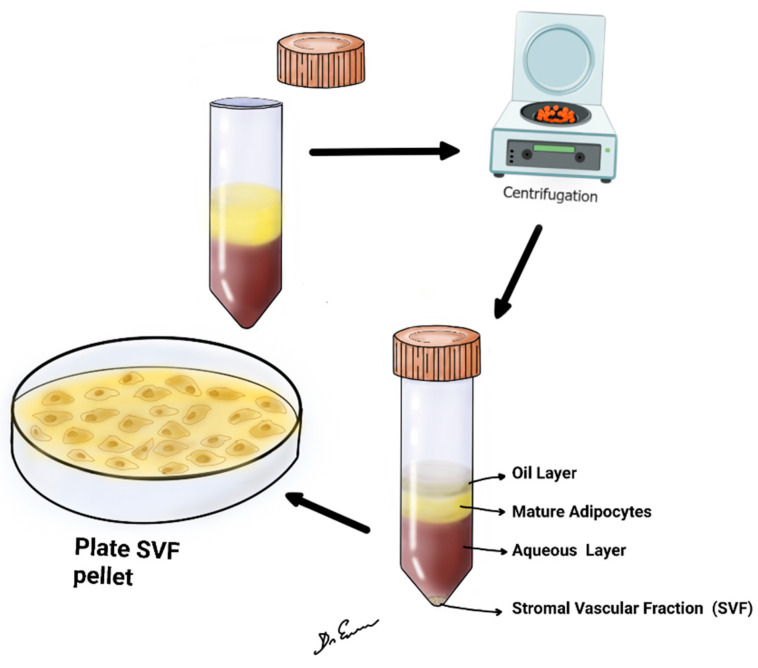
The figure shows lipoaspirate, centrifuged at 2500 or 3000 rpm, for 4 min at room temperature. After centrifugation, upper oil fraction, middle condensed lipoaspirate, lower aqueous fraction, and the stromal vascular fraction were observed.

The reviewed studies collectively demonstrate the potential therapeutic applications of SVF in various medical disciplines. Containing a diverse array of cells such as adipose-derived stem cells (ADSCs), pericytes, and smooth muscle cells, the SVF has demonstrated promising regenerative, immunomodulatory, and anti-inflammatory effects (Figure 1). Characterizing the purification of SVF is of paramount importance, especially when considering its application in therapeutic contexts. The purification process ensures that unwanted components, potentially harmful contaminants, or non-functional elements are removed, leaving behind a highly enriched fraction that can be safely and effectively used for regenerative purposes [3]. The purification and analysis of SVF entail a comprehensive evaluation of its cellular and molecular constituents. First, cellular composition is often deciphered using flow cytometry, which uses specific markers to quantify cell types, such as ASCs (CD34+, CD31−, CD45−), endothelial cells (CD31+), and immune cells (CD45+). Additionally, microscopy, such as histological or fluorescent examinations, visually presents cellular composition [3,4,27,28,29,30,31,32,33,34] (Table 2).

In the field of orthopedics, SVF has been investigated for the treatment of osteoarthritis (OA). In a retrospective study by Kim et al. [12] with 43 participants, SVF implantation was found to improve pain and cartilage regeneration in knee OA. Garza et al. [15] reported improved pain scores and cartilage regeneration in patients with knee OA following SVF implantation, and both studies underscored the absence of complications. Furthermore, Zhang et al. [13] demonstrated that SVF treatment resulted in better clinical outcomes compared to hyaluronic acid therapy in knee OA patients. In the retrospective study conducted by Brian et al. [20], which involved 350 participants, a pivotal focus was placed on patients with arthritis undergoing SVF cell therapy. This study stands out for its significant findings, which revealed marked improvements in both pain levels and mobility among the treated patients, especially notable in those diagnosed with stage III arthritis. These improvements were not just incremental but substantial, indicating a pronounced therapeutic effect of SVF therapy on the symptoms of arthritis [20]. In a prospective study by Perdomo-Pantoja et al. [17] with 36 participants, SVF was found to be comparable to bone marrow cells (BMCs) in spinal fusion, suggesting its viability in spinal surgeries. Similarly, Choi et al. [18] demonstrated a higher early bone fusion rate when using SVF, pointing towards its potential in enhancing spinal fusion outcomes in their prospective study with 10 participants. In plastic and reconstructive surgery, SVF-enriched fat grafting has shown positive results in improving graft survival and wound healing, as reported by Onoi et al. [11]. The prospective study by Kwon et al. [14], involving 20 participants, reported improved outcomes in scar revision surgery following SVF treatment. The study did not report any complications.

Moon et al. [21] explored the use of SVF in reconstructive surgery, particularly for nasal defect repair. The study demonstrated that tissue-engineered dermis grafts incorporating SVF yielded superior scar quality in the alar zone of the nose compared to traditional artificial dermis grafts. This finding is significant as it highlights SVF’s potential in improving aesthetic outcomes in reconstructive surgery, offering more effective solutions for challenging areas like nasal defects [19,21]. Jeon et al. [24] reported increased fat graft survival rates in breast reconstruction with SVF. These findings suggest that SVF has broad applications in the field of regenerative medicine.

Cardiology is another area where SVF therapy is being explored. Pre-clinical and early-phase clinical trials have shown encouraging results in the use of SVF for myocardial ischemia, as mentioned by Bai et al. [9]. This highlights the potential of SVF in cardiac regenerative medicine, although further research is needed to establish its safety and efficacy in larger clinical trials.

It is worth noting that the reviewed studies have reported generally positive outcomes and a favorable safety profile for SVF therapy. Complications were infrequently reported across the studies, indicating a relatively low risk associated with SVF treatments. However, it is important to interpret these findings with caution due to the limited number of participants in some studies and the lack of long-term follow-up data [25]. Despite the promising results, this review also highlights the need for further research to address the limitations and challenges associated with SVF therapy [35,36,37,38,39] (Table 3).

For instance, the variability in SVF composition and preparation methods, as well as the optimal dosage and delivery methods, warrant further investigation. The standardization of protocols and rigorous clinical trials will be crucial for establishing the safety and efficacy of SVF therapies. The findings indicate positive outcomes in terms of pain reduction, tissue regeneration, and improved clinical efficacy. However, further research is needed to address the limitations and challenges in order to unlock the full therapeutic potential of SVF and establish its role as a mainstream treatment option in regenerative medicine.

Despite the limitations of some studies due to small sample sizes, the overall findings support the potential of SVF as a versatile tool in regenerative medicine. However, further research is necessary to address challenges such as standardizing SVF isolation and processing methods, optimizing dosage and delivery approaches, and conducting long-term follow-up studies to assess durability and potential side effects. The therapeutic potential of SVF is far from being fully realized, and there are still many avenues to explore in order to unlock its complete potential. As highlighted in this review, the current body of research supports SVF’s role in regenerative medicine, but it also highlights the need for further study to explore its full potential and tackle existing challenges. Future studies should aim to elucidate the precise mechanisms of action of SVF in tissue repair and regeneration. Our understanding of the therapeutic effects of SVF has been primarily attributed to the ADSCs; however, SVF is a heterogeneous cell population, and the roles of the other cellular components remain relatively unexplored. Understanding the individual and synergistic roles of all the cellular components of SVF could lead to the development of targeted and personalized therapeutic strategies [40,41]. The standardization of SVF preparation methods is an important area that requires attention. Current studies have utilized a variety of protocols for the extraction and processing of SVF, leading to a wide variation in the cellular composition and concentrations. Therefore, the standardization of protocols for SVF isolation and processing will be essential to ensure the reproducibility of results across different studies and clinical settings [42].

### 4.1. Delivery Methods

Dosage and delivery methods are also aspects that need further investigation. The optimal dose and the best route for administration that would maximize the therapeutic effect while minimizing potential adverse reactions are yet to be determined [43]. The route of administration for stromal vascular fraction (SVF) depends on the therapeutic target: it can be directly injected into joints for orthopedic conditions, applied to wounds for healing, introduced intravenously for systemic diseases, delivered into the spinal canal for neurological disorders, or even injected into muscle tissues or brain tissue for specific conditions. The chosen route is always based on the condition in question and ongoing research [44]. Karina et al. [45] showed that the administration of a high dose of SVF up to 10 billion cells in a majority of 421 patients through infusion, spinal, and intra-articular injection was feasible without causing major adverse events and should be further investigated in well-designed phase I-II clinical trials to address the safety and efficacy of the therapy.

Additionally, comprehensive long-term follow-up studies are needed to assess the durability of the therapeutic effects of SVF and to monitor for potential side effects or complications. While the reviewed studies generally report a favorable safety profile for SVF, long-term data will be crucial in solidifying these initial findings [46].

The use of SVF in regenerative medicine signifies a novel and promising approach with potential applications across a broad range of medical disciplines. With its potent regenerative, immunomodulatory, and anti-inflammatory effects, SVF has demonstrated promising outcomes in orthopedics, cardiology, plastic and reconstructive surgery, and more [39]. Importantly, the evidence presented in the reviewed studies suggests a generally favorable safety profile for SVF therapy, marking an encouraging advance in regenerative medicine [47]. These positive outcomes support the potential of SVF as a versatile therapeutic tool, even though further research is needed to fully realize its potential and translate the findings into routine clinical practice.

In a study conducted from 2016 to 2019, Cai et al. [48] evaluated the efficacy of SVF gel in treating chronic wounds. The results highlighted a 100% wound closure rate within an average of 28.3 ± 9.7 days and no recurrences during a 2- to 3-year follow-up. Mechanistic examinations suggested the role of certain growth factors in enhancing cell proliferation and migration, especially in serum-free conditions [49]. Several challenges, however, remain. Standardizing SVF isolation and processing methods, optimizing dosage and delivery methods, and long-term follow-up studies are areas that require further exploration. Zhang et al. [13] investigated the mid-term prognosis of SVF treatment for knee osteoarthritis during a minimum of 5 years, showing that the SVF group had superior VAS and WOMAC scores, and indicated enhanced pain management and knee functionality compared to the HA group. Additionally, SVF showcased a prolonged effectiveness of 61.5 months compared to Na’s 30.3 months. Notably, SVF reduced the risk of clinical failure by 2.6 times, with BML severity and BMI identified as independent prognostic factors. Moreover, while both treatments saw a decline in cartilage volume, the reduction was less pronounced in the SVF group, suggesting potential cartilage protective effects [13]. Additionally, a deeper understanding of the precise mechanisms through which SVF contributes to tissue repair and regeneration is crucial. Different extraction methods and protocols have been developed to harvest SVF, each with its unique strengths and limitations tailored to specific clinical requirements.

Enzymatic Digestion using Collagenase: This technique is particularly known for its efficiency in yielding a high number of SVF cells. By mincing and then digesting adipose tissue with collagenase, the embedded SVF cells are released. Though widely recognized and practiced, this method does raise concerns, especially pertaining to the potential contaminants introduced by animal-derived collagenase. Variations in enzyme quality can also be a bottleneck, sometimes leading to inconsistent outcomes [50]. Given these potential risks, certain regulatory bodies may have reservations about its applicability, especially in human therapeutics [51].

Mechanical Methods: By leveraging physical forces, such as shaking or ultrasonication, SVF cells are extracted from the adipose matrix. This technique’s hallmark is its enzymatic-independent approach, making it favorable in regions with rigorous clinical regulations, as there is no risk of enzyme-related contamination [52]. However, the trade-off includes a comparatively lower cell yield and the potential mechanical stress on the cells, which could compromise their viability.

Water Jet-Assisted Liposuction: A more contemporary method, this technique employs high-pressure water jets to dissociate SVF from adipose tissue. Its minimally invasive nature is its most notable feature, potentially reducing patient discomfort and procedure duration [53]. However, this method comes with caveats, including the need for specialized equipment and expertise. Additionally, the high-pressure jets might inadvertently cause cell damage, raising questions about the viability of the harvested cells [54].

### 4.2. SVF Preparation Steps

The steps of SVF separation can be summarized as (a) liposuction, (b) mechanical separation or shredding, (c) initial filtration, (d) washing, (e) final filtration, (f) SVF and adipose graft harvesting, and (g) cell counting and/or characterization (Table 4) [51,52,53,54].

As we continue to explore and understand the individual and synergistic roles of all cellular components within SVF, the development of targeted and personalized therapeutic strategies becomes a tangible possibility. Considering the rapidly evolving research landscape, large-scale, randomized clinical trials will play a pivotal role in firmly establishing the safety and efficacy of SVF therapies [44]. This will help determine the most effective way to integrate this novel therapeutic approach into mainstream medical practice. While we have only just begun to scratch the surface of the potential applications of SVF in regenerative medicine, the results thus far are encouraging. Medical devices for the preparation of AD-SVF are summarized in Table 5 [43,44,45,46].

The coming years promise to shed more light on this versatile therapeutic tool, and it is our hope that the relentless pursuit of knowledge in this area will usher in a new era of regenerative medicine, leading to improved patient outcomes across a myriad of health conditions [75,76,77].

### 4.3. Immediate Expectations

Increased clinical trials: An increase in clinical trials is anticipated, targeting the efficacy and safety of SVF across various therapeutic applications. These trials are expected to provide critical data that will inform clinical practice and further research, particularly in areas such as osteoarthritis, wound healing, and myocardial ischemia or neurosurgery.

Technological advancements: The immediate horizon also sees advancements in the technology used for SVF extraction and purification. Efforts will likely be directed toward standardizing protocols to improve the viability and potency of harvested cells, which is essential for ensuring consistent and effective treatment outcomes. Regulatory processes for SVF-based therapies are expected. These advancements will facilitate the transition from laboratory research to clinical applications, ensuring that new treatments are safe and compliant with regulatory standards.

### 4.4. Long-Term Expectations

Broad-spectrum applications: Over the long term, SVF is expected to find applications in broader medical disciplines. This expansion could offer novel treatments for various chronic diseases and degenerative conditions and in the flourishing field of tissue engineering.

Personalized medicine and integration with other therapies: Future research might enable the use of SVF in personalized regenerative therapies tailored to individual patient needs and specific conditions. This approach could significantly enhance the efficacy of treatments and minimize potential side effects. There is potential for SVF to be combined with other regenerative approaches, such as gene therapy or 3D-bioprinting [78,79,80,81]. This integration could enhance therapeutic outcomes and pave the way for more comprehensive treatment strategies.

### 4.5. Future Research Directions

Clarifying cellular dynamics: future studies should focus on the specific roles of different cell types within SVF and their synergistic effects in tissue repair and regeneration. Understanding these dynamics is critical to maximizing the therapeutic potential of SVF.

Long-term clinical studies: conducting studies with extended follow-up periods is crucial. These long-term clinical trials are necessary to assess the efficacy and safety of SVF-based therapies over time and to understand the lasting impacts of these treatments.

Dose–response relationship: investigating the optimal dosage and administration routes for SVF in various clinical conditions is essential. This research will help in determining the most effective treatment protocols.

Mechanistic studies: delving into the molecular pathways influenced by SVF can provide deeper insights into its regenerative mechanisms. This knowledge is pivotal for developing targeted therapies to address specific medical conditions more effectively [75].

It is imperative to address ethical concerns and develop comprehensive regulatory guidelines for using SVF in clinical settings. These guidelines will ensure that treatments are practical, ethically sound, and compliant with legal standards.

### 4.6. Limitations of this Study

Lack of long-term data: many studies may have had short follow-up periods, limiting the ability to draw conclusions about the long-term safety and efficacy of SVF.

Limited sample size: this review may be constrained by the small sample sizes of some included studies, reducing the power to detect significant effects.

Regulatory landscape: differences in regulatory practices across countries may affect the applicability and generalizability of this review’s findings.

## 5. Conclusions

Our literature review on SVF provides valuable insights into its potential as a powerful tool in regenerative medicine. SVF, composed of a heterogeneous mixture of cells including ADSCs, has demonstrated significant therapeutic efficacy and safety in various medical disciplines. The reviewed studies highlight the positive outcomes of SVF therapy in areas such as orthopedics, plastic and reconstructive surgery, cardiology, and wound healing. SVF has shown promising results in reducing pain, improving tissue regeneration, enhancing graft survival, and promoting wound healing. Moreover, SVF has exhibited immunomodulatory and anti-inflammatory properties, contributing to its regenerative effects. The future of SVF in regenerative medicine holds great promise. Continued research, technological advancements, and regulatory guidelines will contribute to unlocking its full therapeutic potential. The standardization of protocols and large-scale clinical trials will provide robust evidence and establish SVF as a mainstream treatment option. With these developments, SVF has the potential to revolutionize the field of regenerative medicine and offer innovative solutions for a wide range of medical conditions. SVF represents an exciting and evolving field of research that has the potential to transform the landscape of regenerative medicine. By harnessing the regenerative and immunomodulatory properties of SVF, researchers and clinicians can pave the way for innovative treatments that improve patient outcomes and quality of life.

## Figures and Tables

**Table 1:** Data Synthesis and Analysis.

Author(s) (Year)	Study Type	Sample Size	Key Findings	Conclusion	Complications
Onoi et al. [11] (2023)	prospective case series	42	Safety of autologous SVF	SVF cell injections in the hip joint showed good short-term clinical efficacy for reducing hip OA symptoms.	no
Kim et al. [12] (2023)	retrospective	43	Cartilage repair was evaluated based on the Magnetic Resonance Observation of Cartilage Repair Tissue scoring system, using the magnetic resonance imaging from the 12-month follow-up	SVF implantation improved pain and cartilage regeneration for patients with knee osteoarthritis. The cartilage lesion size and the number of SVF cells significantly influenced the postoperative outcomes.	no
Zhang et al. [13] (2022)	retrospective, randomized controlled clinical trial	126	The VAS and WOMAC scores in the SVF group were significantly better than those in the hyaluronic acid group during the 5-year follow-up after treatment.	Up to 5 years after autologous SVF treatment, acceptable clinical state was present for approximately 60% of patients with less cartilage volume loss. In addition, the high severity of BML and high BMI increased the risk of clinical failure. Intra-articular injections of SVF do not improve subchondral BML.	no
Kwon et al. [14] (2023)	prospective	20	The 6-month follow-up following scar revision surgery revealed better results after treatment with SVF than those in the control group.	Although more research is needed, autologous SVF is a valuable source of regenerative medicine that can be swiftly and inexpensively prepared from human fat tissue.	no
Garza et al. [15] (2021)	prospective double-blinded randomized trial	39	The median percentage change in WOMAC score at 6 months after injection for the high-dose, low-dose, and placebo groups was 83.9%, 51.5%, and 25.0%, respectively. The high- and low-dose groups had statistically significant changes in WOMAC scores when compared with the placebo group (high dose, *p* = 0.04; low dose, *p* = 0.02). The improvements were dose-dependent.	Intra-articular SVF injections can significantly decrease knee OA symptoms and pain for at least 12 months. The efficacy and safety demonstrated in this placebo-controlled trial support its implementation as a treatment option for symptomatic knee OA. Magnetic resonance image review revealed no changes in cartilage thickness after treatment.	no
Rodriguez-Merchan et al. [16] (2022)	literature review	28	Intra-articular injection of SVF seems to be a safe and efficacious method for managing knee osteoarthritis (OA). Platelet-rich plasma (PRP) and SVF are safe and effective management for intractable Achilles tendinopathy in humans, although subjects treated with SVF recover earlier.	The SVF can safely be used to treat diabetic subjects suffering from chronic foot ulcers. Experimental studies indicate that SVF could be a new option to osseous regeneration.	no
Perdomo-Pantoja et al. [17] (2021)	prospective	36	The aim of this study was to compare the efficacy of freshly isolated adipose tissue-derived stromal vascular fraction (A-SVF) cells and bone marrow cells (BMCs) in achieving spinal fusion on rat models.	SVF cells yielded a comparable fusion mass volume and radiographic rate of fusion to BMCs when combined with a clinical-grade bone graft substitute. These results suggest the feasibility of using freshly isolated A-SVF cells in spinal fusion procedures.	no
Choi et al. [18] (2020)	prospective	10	Two polyetheretherketone (PEEK) cages were inserted into the intervertebral space following the complete removal of the intervertebral disc. The PEEK cage (SVF group) on the right side of the patient was filled with β-TCP in combination with SVF, and the cage on the left side (control group) was filled with β-TCP alone. Fusion rate and cage subsidence were assessed by lumbar spine X-ray and CT at 6 and 12 months postoperatively. At the 6-month follow-up, 54.5% of the SVF group (right-sided cages) and 18.2% of the control group (left-sided cages) had radiologic evidence of bone fusion (*p* = 0.151).	The 12-month fusion rate of the right-sided cages was 100%, while that of the left-sided cages was 91.6% (*p* = 0.755). Cage subsidence was not observed. Perioperative combined use of SVF with β-TCP is feasible and safe in patients who require spinal fusion surgery, and it has the potential to increase the early bone fusion rate following spinal fusion surgery.	no
Rowe et al. [19] (2023)	prospective	344	Mesenteric windows from old rats were isolated following exteriorization-induced (EI) hypoxic injury and intravenous injection of one of four cell therapies: (1) SVF from young or (2) old donors, (3) SVF from old donors depleted of or (4) enriched for T cells. Advancing age increased the SVF T-cell population but reduced revascularization following injury.	SVF represents a heterogeneous cell population shown to increase angiogenic regeneration in the researchers’ novel aged mesenteric injury model. This study provides others with a new tool for tracking vascular remodeling and can be used in conjunction with study of cell therapies or drugs in a setting of advanced age. Furthermore, the researchers show how the age of the donor should be considered not only for cellular differences but functionality as a vascular therapeutic. Age-related changes to cell dynamics and function in providing therapeutic gains—that is, the secretion of anti-inflammatory cytokines, increasing sensitivity to VEGF, increasing the migration and engraftment potential of injected cells, and endothelial cell division.	no
Brian et al. [20] (2020)	retrospective	350	Seven days after SVF cell therapy, 45.2% of subjects experienced improved pain levels and mobility. Three, six, and twelve months after therapy, improvement in pain levels reached 75.3%, 84.4%, and 84.9%, and improvement in mobility reached 75.2%, 84.4%, and 84.9%.	The treatment demonstrated a strong safety profile with no severe adverse events or complications reported. The results of the study are showing that SVF cell therapy was more effective in subjects with arthritis stage III compared to arthritis stages I, II, and IV.	no
Moon et al. [21] (2019)	retrospective	77	In the upper two-third and lower one-third zones, except for the ala, no statistically significant differences were found in any parameters. In the alar zone, statistically significant differences were detected in 10 of 21 POSAS parameters.	To cover nasal defects, the tissue-engineered dermis graft may be superior to the artificial dermis graft regarding scar quality at the ala. However, there were no significant differences in other zones.	no
Zimmermann et al. [22] (2018)	retrospective	10	In the transposition group, sustained pain reduction was not observed after an initial significant reduction 2 months post-surgery, resulting in pain relapse at 36 months and pain comparable to the preoperative assessment. In the graft group, some degree of pain reduction was observed at 2 months after the surgery and proved to be constant in the long-term outcome, although not statistically significant compared to preoperative levels.	Both SVF-enriched fat grafting and intramuscular transposition failed to prove statistically significant pain reduction in treating symptomatic neuromas of peripheral nerves.	no
Calcagni et al. [23] (2018)	retrospective	5	Pain reduction observed at 2 months after surgery was constant over time, though not statistically significant compared to preoperative levels.	SVF-enriched fat grafting represents another alternative to numerous available treatments of painful end-neuromas of the SBRN. The researchers’ preliminary results could not show any significant difference in pain reduction following SVF-enriched fat grafting. Further larger trials are required in order to evaluate the therapeutic potential of SVF-enriched fat grafting.	no
Jeon et al. [24] (2021)	prospective	20	Fat graft retention rate was higher in Group 1 than in Group 2 at both postoperative 6 months (73.8% vs. 62.2%; *p* = 0.03) and 12 months (65.4% vs. 48.4%; *p* = 0.03). Group 1 showed higher patient satisfaction. Regarding complications, fat necrosis occurred in one patient in each of the two groups. However, locoregional recurrence was not observed in any patient during follow-up.	CAL with SVF is effective in increasing survival rates of autologous fat grafts for correction of volume deficit after breast reconstruction. Moreover, it is associated with improved patient satisfaction in terms of the aesthetic aspect.	fat necrosis occurred in one patient
Yin et al. [25] (2021)	prospective	5	All patients were treated with surgical debridement, cell suspension (SVF cells suspended by platelet-rich plasma) injection into the wound, and platelet-rich plasma gel coverage. Wounds were measured every week after treatment using a two-dimensional digital camera and a three-dimensional wound measurement device. All patients were followed-up for 4 months after the treatment.	The average proportion of granulation tissue achieved 100% within 4 weeks for all cases. The wound size decreased to less than half of the original size for all cases 4 weeks after the treatment. Findings revealed that the new treatment is efficient to achieve wound healing in patients with recalcitrant chronic diabetic ulcer of lower limb.	no
Aletto et al. [26] (2022)	prospective clinical trial	123	One single injection of lipoaspirate reduces knee pain and improves function after 1 month from the injection.	The intra-articular knee injection of SVF is safe and effective to ameliorate the clinical and functional scores in patients with early knee osteoarthritis for 6 months.	no

**Table 2:** SVF cell content isolated from the aqueous portion.

Type of Cells	Functions	Authors, Year [ref.]
Mesenchymal progenitor/stem cells	They have the capacity to perform self-renewal and differentiation into specific cell lineages, and support the maintenance of other cells via paracrine secretion.	Francis et al., 2018 [27,32]
Lymphocytes	They participate in both innate and adaptive immune responses with multiple effect or functions. They produce antibodies, direct the cell-mediated killing of virus-infected and/or tumor cells, and regulate immune responses.	Busato et al., 2020 [28]
Smooth muscle cells	They display involuntary contractile activity to control the diameter, wall movement, and wall stiffness of specific organs.	Guimarães, 2017 [29]
Adipose tissue-derived stem cells	They secrete growth factors, cytokines, and antioxidant factors into a microenvironment, regulating intracellular signaling pathways in neighboring cells. Protective outcome via anti-inflammatory and immunomodulatory effects.	Bora et al., 2017 [3]
Preadipocytes	They promote the growth of adipose tissue by differentiating into mature and metabolically active adipocytes. Proliferating preadipocytes may also exhibit phagocytic activity towards microorganisms and behave similarly to macrophage-like cells.	Matsuo et al., 2020 [30]
Mφ2 macrophage	The type 2 macrophage (Mφ2) is produced by the type 2 T helper immune response and takes on an anti-inflammatory role, typically characterized by an increase in the production of interleukins (IL-4, IL-5, IL-9, and IL-13). It is also directly involved in regenerative and tissue repair processes that occur after injuries.	Contreras et al., 2015 [31]; Dey et al., 2021 [32]
T cells	As components of the adaptive immune system with major importance, these cells are responsible for eliminating infected host cells, activating other immune cells, and secreting cytokines that further regulate immune responses.	Dulong et al., 2022 [33]
Endothelial precursor cells and endothelial cells	They differentiate into functional endothelial cells and sustain vasculogenesis by incorporating themselves into the injured endothelium with the formation of functional blood vessels and through the local secretion of pro-angiogenic factors, with a paracrine effect on the cells that form the vessel. They play a critical role in vascular homeostasis as well as physiological or pathological processes such as thrombosis, inflammation, and vascular wall remodeling. Resting endothelial cells control blood flow and the passage of protein from blood into tissues, as well as inhibiting inflammation and preventing coagulation.	Gulyaeva et al., 2019 [34]

**Table 3:** Effect of stromal vascular fraction on tissues.

Regulation of pro-inflammatory molecules	Decreases IL-1b and IL-6 levels.
Hyaline cartilage extracellular matrix	Increases Glycosaminoglycan level.
Triggering of IL-1Ra	Reduces the catabolic effect of IL-1.
Increasing of ADAMTS-4 and -5	Provides tissue balance (homeostasis).
Anti-inflammatory	Reduces tissue swelling (edema).
Anti-apoptotic	Reduces and stops programmed cell death.
Increasing of TIMPs-1, -3, and -4 metalloproteinases	Provides tissue balance (homeostasis).

ADAMTS, A disintegrin and metalloproteinase with thrombospondin motifs; TIMPs, tissue inhibitors of mMetalloproteinases.

**Table 4:** Steps of stromal vascular fraction separation.

	Conventional	Modified Approach
Obtaining adipose tissue	- Abdominal fat - Reusable Sorenson-type lipoaspiration cannula - Klein’s Translumination solution: Modified - Klein solution (500 mL isotonic, 20 mL lidocaine, 2% epinephrine, 2 mL bicarbonate) - 50 mL Luer lock syringe	- Abdominal fat - Disposable/Re-usable Coleman-style cannula - Klein’s Translumination solution: Modified - Klein solution (500 mL isotonic, 20 mL lidocaine, 2% epinephrine, 2 mL bicarbonate) - 50 mL Luer lock syringe
Mechanical separation/shredding	- Shredding of tissue by shaking with glass ball (shaking time and strength depend on the user)	- Separation by the effect of gravity in a screw-form mechanical separator at standard power and time
Pre-filtration	- Polyethylene filtration in a 100 micrometer porous polyethylene bag	- Filtration with the effect of gravity in a 100-micrometer porous device whose base is supported by a metallic or polymeric cage
Washing	- Not available	- Washing in the device
Final filtration purity	- Filtration on 10-micrometer porous polyethylene filters in 10 mL syringes	- Final filtration with the rise of adipose tissue and SVF to the solution surface in serum within the device
Collection of SVF/adipose tissue	- Available in an equivalent system	- Proximal adipose tissue and SVF separation reservoir
Cell counting and characterization	- Cell counting, determination of viability, determination of cell characteristics, and histochemical identification	- Cell counting, determination of viability, determination of cell characteristics, and histochemical identification

**Table 5:** Commercial medical products for AD-SVF preparation [43].

Product	Company	Article
Cha-Station	Somnotec http://www.somnotec.net (accessed on 15th Novembre 2023)	[55]
Octagone D200	Endecotts Ltd. https://www.endecotts.com (accessed on 15th Novembre 2023)	[56]
AdiPrep	Harvest http://www.harvest.co.kr/clinician/clinician-home/adiprep/advantages/quality.html (accessed on 15th Novembre 2023)	[57]
Lipokit	Medi-Khan http://www.medikanint.com (accessed on 15th Novembre 2023)	[58,59]
Puregraft 250	Puregraft LLC http://www.puregraft.com (accessed on 15th Novembre 2023)	[60]
Lipogems	Lipogems http://understandlipogems.com (accessed on 15th Novembre 2023)	[61,62]
MyStem	MyStem LLC https://mystem.eu/ (accessed on 15th Novembre 2023)	[63,64]
Arthrex SVF	https://www.arthrex.com/orthobiologics (accessed on 15th Novembre 2023)	[65]
Adinizer	BSL http://biosl.com/?ckattempt=1 (accessed on 15th Novembre 2023)	[66]
Microlyser	Tlab https://tlab.com.tr/en/products/microlyzer-svf-kit/ (accessed on 15th Novembre 2023)	[67]
SEFFIE	Advanced-Maes http://www.advanced-maes.com/ (accessed on 15th Novembre 2023)	[68]
LIPOCUBE	STEMC https://lipocube.com/ (accessed on 15th Novembre 2023)	[69,70]
Q-Graft	Human Med AG https://www.humanmed.com/en/products/q-graft/ (accessed on 15th Novembre 2023)	[71]
Tulip Nanotransfer	Tulip Medical https://tulipmedical.com/ (accessed on 15th Novembre 2023)	[72]
Lipocell	Tissyou https://www.tissyou.com/portfolio_page/lipocell/ (accessed on 15th Novembre 2023)	[73]
LipiVage	Genesis Biosytems https://www.genesisbiosystems.com/lipivagesystem-autologous-fat-transfer/ (accessed on 15th Novembre 2023)	[74]
